# Assessing the impact of sand and dust storm on agriculture: Empirical evidence from Mongolia

**DOI:** 10.1371/journal.pone.0269271

**Published:** 2023-02-06

**Authors:** Hayatullah Ahmadzai, Arzoo Malhotra, Seta Tutundjian

**Affiliations:** Research and Innovation Department, International Center for Biosaline Agriculture (ICBA), Dubai, United Arab Emirates; North Carolina State University, UNITED STATES

## Abstract

Assessing the economic impact of sand and dust storms provides critical insights to policy development and reforms; a subject that is gaining more attention as risk management becomes the dominant approach for hazard mitigation policies. To assess the causal impact of sand and dust storms on agriculture, specifically on crop and livestock revenue and physical production, random year-to-year variations in dust exposure were analyzed using a fixed effect regression. To complete this analysis, weather and climate data from the on-ground meteorological stations was combined with the household level socioeconomic surveys conducted by Mongolia’s National Statistics Office (NSO) over a decade. The descriptive statistics of the meteorological data collected over the eight years period show that, on average, 29 dust events have occurred every year across the country, with greater variation among provinces (Aimags) and regions, reaching up to 108 events in a year in some provinces. The overall trend reveals a slight decrease in the dust events from 2009 to 2019. The econometric results show that value of crop and livestock production (gross income) and physical yields significantly decline in response to higher frequencies of sand and dust storms events. During this period, Mongolia experienced a 2.7% decline in crop revenue as a result of additional sand and dust storms. Assuming 2.7% constant decline in revenues across all agricultural sub-sectors and regions or Aimags, this could lead to about $37.8 million in losses to the economy, which is equivalent to about 0.27% of the national GDP of Mongolia. Increases in the frequency of sand and dust storms could reduce agricultural productivity by between 1.5% to 24%, depending on the crop. Estimates from the modelling exercise are robust to potential endogeneity bias in the measure of sand and dust storms; different specification and identification approaches accounting for the endogeneity bias consistently reveal negative and qualitatively similar impacts of sand and dust storms on crop and livestock productivity.

## Introduction

Sand and dust storms (SDS) present a formidable challenge to achieving sustainable development in its threethree dimensions—economic, social, and environmental [1]. SDS often demolish critical agricultural assets and infrastructure, distort production cycles, trade flows, and livelihoods generation [2]. Studies in the ‘dust belt’ through North Africa, the Middle East, Asia, and China over the past two decades found significant impact from SDS on farmer incomes across the entire region (Gholizadeh et al, 2021) [3]. The impacts of SDS threaten global food security and cause additional disruptions throughout food value chains, particularly in communities where agriculture and food production account for a large share of income, employment, and food security, and nutrition. Given the increasing threats posed by the natural hazards and climate change to livelihoods, environment, and the economy, these will further challenge the implementation of a broad range of the Sustainable Development Goal (SDG) targets. SDS have been identified as a high priority issue; the United Nations General Assembly (UNGA) adopted resolutions entitled “combatting sand and dust storms” in 2015 (A/RES/70/195) and 2016 (A/RES/71/219). These resolutions recognize and acknowledge that sandstorms and dust storms represent a critical impediment to sustainable development in severely affected countries and regions and urge individual countries to address the challenges posed by them through appropriate policy measures. The United Nations Convention to Combat Desertification (UNCCD) is playing a leading role in addressing hazards posed SDS to assist vulnerable countries to develop policy with a main focus on disaster risk reduction, as advocated by the Sendai Framework [4, 5].

An estimated two billion tonnes of fine particles are raised by winds from the world’s dryland soil surfaces every year [6] affecting 151 countries directly by sand and dust storms The sources of these dust storms areas falls in 45 countries of which 38 are in Africa and Asia [5]. Over the past few decades, research work has highlighted the devastating effects of sand and dust storms and environmental emissions on important outcomes, from health to economic growth and development. Research also points to the risk hotspots and areas where the recorded sand and dust storm events have increased over the past decades [7–9].

SDS effect crop production in several ways, from germination to growth and development. The most damaging impact of SDS on crops is the direct loss of plant tissue due to sandblasting by sand and soil particles. The loss of plant tissue reduces photosynthetic activity, inhibiting the production of energy needed for growth and reproduction, thereby inhibiting the development of grain, fiber, or fruits [2]. The magnitude and extent of impact of SDS on crops greatly depends on the growth stage of the plants. If the low severity SDS events occur early in the plant’s growth cycle, losses may be minor if plants have time to regrow lost leaves. However, in the event of extreme SDS occurrences early in the growing season, young plant could be buried and possibly completely destroyed due to the lack of sunlight for photosynthesis, forcing farmers to re-sow their fields [10, 11]. SDS events occurring later in the growing season could reduce yield during the critical grain development phase; damages right before harvest could result in direct harvest loss. Middleton et al., (2018) noted that the impact on perennial crops could be similar to that on annual crops, in that the current year output could be lost or reduced. However, perennial plants may also experience long-term damage, thereby reducing yields far beyond the occurrence of the SDS event. Moreover, sand and dust storms driven erosion effects crop productivity on long-term basis by removing the topsoil layer rich in nutrients and organic matter [2]. The erosion of this layer exacerbates soil erosion and accelerates the process of land degradation and desertification, thereby perpetuating the cycle of increased SDS frequencies. Other impacts of SDS on agriculture include damages to key agricultural infrastructure, including blocking of irrigation canals with sediments, covering transportation routes with particles, soiling water sources, and polluting the atmosphere.

Although wider effects of SDS are widely documented in the literature, their quantitative losses and impact on agriculture and other economic activities do not feature prominently in available development and economic literature [11]. Little empirical evidence exists to inform policy of the harmful effects of SDS on the agriculture industry. Lack of data on SDS and agriculture output remains a major challenge for empirical research to quantify losses attributed to SDS in agricultural sub-sectors. Additional challenges to data collection are encountered due to the discrepancy between agricultural and climate data in terms of measurement and coverage. While most data collected on SDS is point-based, generally collected by remote sensing and satellite imagery or meteorological stations, data on agriculture outputs is usually collected at the household or sector level. This mismatch and inconsistency in the measurement and coverage of data makes it empirically difficult for researchers to systematically quantify the actual losses in agriculture production and farm revenues associated with SDS.

Assessing the impact of SDS on economic activities, including agriculture, provides critical insights to inform future policy development and reforms, especially as risk management becomes the dominant approach of hazard mitigation policies [5]. This research is intended to facilitate the development of effective mitigation policies in Mongolia, which could also be applied to other countries with similar agro-ecological and economic contexts that also suffer from persistent dust emissions. This paper presents empirical evidence analyzing the impact of dust exposure on crop production in Mongolia. The analysis spans historical household-level and climate data, allowing for a thorough exploration of the effect of SDS on crop productivity, physical yields of various crops, land use, and vegetation cover in Mongolia. Our analysis reveals compelling evidence that SDS have significant bearings on both crop and livestock productivity, ground biomass, and was found to reduce crop revenues by 3% and crop yields by 1.5–24% depending on the crop type.

## Dust storms in the context of Mongolia

Dust storms in Mongolia affect the entire country, with most activity occurring in the spring in the southern Gobi region [12]. Over 70% of the storms occur in dry soil conditions [13]. A large portion of Mongolia is occupied by the Gobi desert plains in southeast Mongolia [14]. Over these vast areas, dust storms are frequent and sometimes severe, especially in late spring to early summer. “Ugalz”, the native term used for dust storm, can be translated as spiral or whirl, and is problematic for agriculturists, animal herders, and breeders in the Gobi region. The unique topography and geography of the Gobi regions drastically affects the atmospheric weather conditions [12].

While most dust sources assessed are natural, particulate matter can also come from anthropogenic sources. A study on particulate matter of dust storm activity in the Gobi Desert over 16 months in 2009–2010 established that SDS are the result of both natural and anthropogenic sources. Winter months are dominated by particulate matter from coal pollution, while SDS events in the spring are dominated by natural sources due to the passing of cyclones [15]. The impact of humans on erodibility of the land is documented since the 1990s; it is attributed to the harvesting of natural vegetation for fuel wood, overgrazing, and the increased ploughing of steppe land for crop cultivation. Local sources of dust also increased in the 1980s from vehicle movement on unpaved roads, construction work, and pollution from power stations and factories [12].

Using data collected over a 13 year period from 2000 and 2013 from 113 meteorological stations in natural forest steppe, steppe, Gobi Desert, and mountain zones, Amgalan et al., (2017) found that dust event distribution over the country exhibits a heterogeneous spatiotemporal pattern. The highest number of dust storm days in south-eastern and western parts of Mongolia were identified as the primary sources of SDS. These findings are consistent with an earlier study [13] that studied dust activities in Mongolia using a composite of synoptic data between 1937 and 1999, and concluding that the dust-affected regions in Mongolia are mainly its southern and western parts: the area of Great Lakes depression, the desert and the steppe-desert, and Mongol Els area of west Mongolia. The highest frequency of dust storms were over the three areas in the Gobi Desert in Mongolia, specifically, the south side of the *Altay Mountains* and around *Ulaan-nuur Lake* and *Zamiin-Uud*. Similar conclusions were confirmed by other studies that the highest frequency of SDS occurs in southeast Mongolia, with a rate of about 20–50 times per year [16].

While most regions of China experienced a decrease over 34 years, the frequency of SDS events increased in Mongolia and northern inner Mongolia between 1998 and 2007 [17]. This increase was associated with worsening drought conditions and the corresponding decreases in precipitation, as well as reduced surface vegetation and soil moisture after the mid-1990s. Overgrazing along with intensifed human activities such as coal mining has contributed to the widespread land degradation in Mongolia, while climate change has become a major driving factor for recurring droughts [18].

Precipitation rates across Mongolia greatly vary throughout the year leading the interannual variations in the sand and dust storm events. Previous studies on Mongolia suggested that the occurrence of dust events exhibit considerable interannual variations [19]. According to their research, the occurrence of SDS events largely depends on the amount of annual precipitation. Dusty days in high dust-frequency years were associated with strong wind days with precipitations of about 10 mm. Conversely, SDS occurrences were suppressed by high precipitation (approximately 22 mm or greater) in dust-less years over the south-eastern part of the Gobi Desert in the month of May.

## Estimation strategy and identification

Broader literature theorizes the potential damages and impacts of SDS in the agriculture sector. The majority of these studies are qualitative, lacking data and systematic quantitative analysis to assess the true losses of SDS on agriculture. To estimate the causal impact of SDS on crop revenue and production, a fixed effect regression was selected to exploit random year-to-year variation in dust exposure in Mongolia. The analysis largely follow the recent literature on the impact of climate on economy [8, 20, 21], which are also more recently adopted by researchers analysis the impact of sand and dust storms [22, 23]. Our econometric specification benfits from the previous literature and we construct a standard panel model to estimate the following Eq:

$$Y_{st}=\alpha +\sum_{k=0}^{j}\left(\beta_{k}\;SDS_{st}+\gamma_{k}\;C_{st}\right)+W_{t}+L_{s}+\varepsilon_{st}$$ (1)

Where *Y_st_* is an agricultural outcome variable (i.e., production of a crop, farm revenues, land use changes, and vegetation index) in state *s* at time *t*. *SDS_st_* is a weighted measure of the sand and dust storms at state *s* and time *t*, and *β*_1_ is the coefficient of interest to be estimated. *C_st_* is a vector of the control variables that are likely to effect agriculture production. Since weather conditions are important confounders of the dust emission, meteorological factors were controlled for through the inclusion of precipitation and temperature in the estimation. Socioeconomic variables were also included in the analysis, including state-level average agricultural holding and agricultural expenditures; these factors are important drivers of agricultural outputs. In addition, two sets of important fixed effects were factored: wave or year fixed effects, and location fixed effects (i.e., Aimag dummies) to account for variations that evolve over time (i.e., time-specific shocks) and variations attributed to the differences across location in any observable or unobservable predictors. Standard errors are clustered by the province or Aimag dummies to allow for spatial correlations within provinces, which could be attributed to differences in market conditions and government policies.

The panel estimation involving fixed effects allows for control of any potential biases associated with time invariant unobserved determinants of the outcome variables that can be correlated with the weather conditions and other differences in managerial capabilities and practices of farmers in different provinces. Fixed effect estimations delve into the relationship between predictor and outcome variables within each province which involves any shocks common to all provinces (e.g., effects consistent across all provinces), but time-variant are controlled for by including the year fixed effects. Each province has its own individual characteristics that may or may not influence the predictor variables (for example, production systems in each province could have some effect on the agricultural output). Identification may, however, be still challenged by potential endogeneity issues in the predictor of interest.

Potential endogeneity bias in SDS due to time-varying omitted variable bias and reverse causality may present an empirical challenge to identify the effect of interest in the estimation of Eq (1). The biases caused by endogeneity are usually associated with unobserved abilities and motivation. Technically, endogeneity occurs when a predictor variable, in this case, SDS, are correlated with the error term (*ε*) which will lead to an estimation bias in the coefficient of sand and dust storm measure (*SDS_st_*) [24]. This in particular is a major concern, since locally emitted dust might be endogenous to agricultural production due to simultaneity (e.g., the dependent variable and independent variables are jointly determined) and unobserved confounding factors (omitted variable bias). This would mean that in Eq (1), the measure of sand and dust storm (*SDS_st_*) might be correlated to unobserved factors in the error term (*ε*), leading to unrobust estimates. In this case, the baseline model may not be appropriately specified to deliver consistent parameter estimates of SDS. To break this correlation, an instrumental variable is needed to account for the unexpected behavior between these variables. Instrumental variables are variables that are correlated with the SDS measure but must not be collected with the dependent variable (Y). Unfortunately, given the lack of data on candid variables that could potentially serve as instrumental variable is an empirical challenge. Therefore, we recognize this limitation in the present study.

In an attempt to address the endogeneity bias in our estimation, an alternate specification of the baseline model using only non-local dust events was employed as a possible workaround to address potential bias that may affect the robustness of the estimates from the baseline model. Referring to Table 1, externally or non-local emitted dusts are indicated by the WMO’s weather state code 06 originated in areas more than 50 miles away [22]. Non-local dust events are therefore exogenous to the local production and should significantly mitigate the endogeneity bias. A similar identification strategy is followed by a recent study to address the endogeneity bias in their analysis assessing the effects of SDS on firm and agricultural productivity in Iran [22].

**Table 1:** Phenomena related to sand and dust storms based on the WMO weather state codes.

Synop code	Weather description
06	Widespread dust in suspension in the air, not raised by wind at or near the station at the time of observation
07	Dust or sand raised by wind at or near the station at the time of observation, but no well-developed dust or sand whirl, and no dust storm or sandstorm seen
08	Well-developed dust whirls (dust devils) seen at or near the station during the preceding hour or at the time of observation, but no dust storm or sandstorm
09	Dust storm or sandstorm within sight at the time of observation or at the station during the preceding hour
30–32	Slight or moderate dust storm or sandstorm
33–35	Severe dust storm or sandstorm
98	Thunderstorm with dust or sandstorm

## Data and description of variables

There are several sources that provide data to measure dust in atmosphere, including weather stations, satellite imagery and remote sensing, and climate models (reanalysis). We use the Global Met Office Integrated Data Archive System (MIDAS) Land and Marine Surface Stations Data available from the Center for Environmental Data Analysis (CEDA) in the *Center for Environmental Data Analysis, 2021[25]* which provides reliable spatio-temporal data for Mongolia for the period of this study. The dataset used is the most homogenized and hence determined to be the most reliable source of data. There is a precedent for the use of this data in the literature, as it was also used by previous studies to map global SDS [26].

The dataset consists of meteorological records reported at 1- or 3-hour intervals in SYNOP (Surface Synoptic Observations) and METAR (Meteorological Aviation Routine Weather Report) codes. The weather records are reported by either manned or automatic weather stations [26]. Only weather records from the manned SYNOP stations are used to construct the sand and dust storm measures and other climate variables. The World Meteorological Organization (WMO) recognized 11 weather codes relating to wind erosion. The dust event records in the present weather code according to the WMO weather codes are summarized in Table 1 on the following page. We fallow recent studies and use the relevant codes 06, 07, 08, 09, and 30–35 to construct the SDS measure over the study area [26–28]. We take caution and exclude code 98 in the SDS calculation to eliminate the possibility of confusing SDS events with snow events and storms. Inclusion of thunderstorms in the SDS measure given by code 98 may result in an overestimation of SDS’s impact on agricultural outcomes. We take further caution and exclude duplicated records as well as stations that reported weather state codes less than once a day over a month. As for non-local dust events, we use only code 06, as described in the previous section.

**Fig 1:**
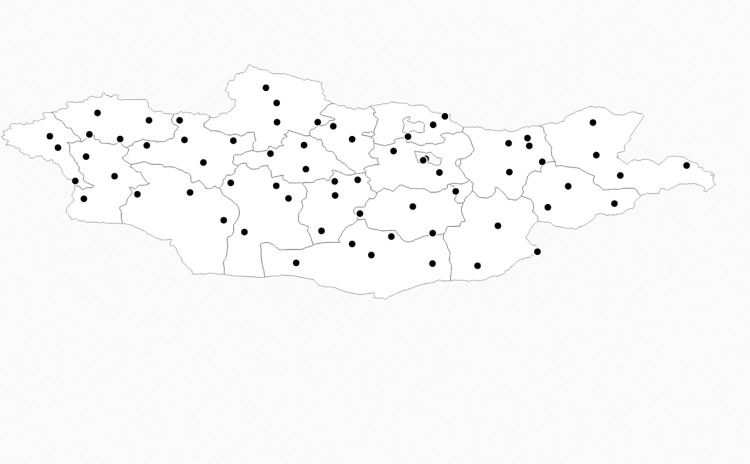
Physical map of Mongolia with weather station locations. Source: Authors’ composition based on MIDAS/CEDA dataset.

Ground weather stations in Mongolia report data on visibility and weather codes on hourly basis for each Aimag or province. The individual measurements classified as sand and dust storm events were counted for each station. The data show that there are single or multiple reports of the weather state code in a given day that qualifies as a sand and dust storm event. If a station reported the relevant weather code once in a day, we extend that state for the whole day. To increase precision, the SDS measure is weighted by the number of available days of observations in a month and/year. We then aggregate the SDS and climate data spatially and across time (i.e., the mean is taken both spatially and temporally) to match our key economic/agriculture variables (from the household surveys), that is, we aggregate the SDS data by aimag-year basis. Moreover, if there are more than one meteorological station located in a particular province or Aimag, we compute an average of the computed SDS measure to estimate a state-year average. Fig 1 shows the location of weather stations across the provinces in Mongolia from which data are obtained overlaid with the key geographic regions and features in the country.

In addition to the present weather state codes, the MIDAS Land and Marine Surface Stations dataset also provides 1- or 3-hourly data on other key climate variables like precipitation and temperature. Hourly records on temperature and precipitation were converted to daily means, which were subsequently used to compute monthly and annual means. Temperature was measured in Celsius, while precipitation was measured in millimeters (mm).

For information on crop yields and revenues, the Household Socio-economic Surveys (HSES) were utilized (The HSES data is publicly available through NSO at http://web.nso.mn/nada/index.php/catalog/HSES/dataset). HSES is a cross-sectional household level data collected by the National Statistics Office (NSO) of Mongolia from 2007/08–2019. The HSES is a nationally representative survey that aims to estimate and monitor the level of poverty of the country and people’s living standards. Geographically, HSES covers about 16,000 households in all Aimags every year. HSES provides data on agricultural revenues and physical production for major crops and other key variables: household characteristics, location characteristics, agricultural landholdings, and agricultural expenditures. The data is reported annuallyand do not identify growing seasons for individual crops. Therefore, the analysis are conducted over the calander year. The household-level data were aggregated to construct average annual aimag-level psudo-panel dataset to match the other data on SDS and climate variables acquired from the CEDA dataset. The aggregation of the data was done by summing the annual values of the individual variable which were then divided by the total land planted to obtain per hectare estimates in each Aimag. These included dependent variables such as revenue and production measures. Production quantities originally reported in kilograms were summed at the Aimag level and divided by the total land cultivated by the household in the respective year to calculate Aimag-level average yields for individual crops. Households also reported annual revenues (gross income) from the sales of crops measured in Mongolian Currency (Togrog). Revenues are also divided by the area cropped to calculate average annual revenues per hectare at the Aimag level. The HSES aimag level data were then merged and appended with the weather data to allow for econometric analysis to assess the impact of SDS on agricultural sub-sectors. The data were log-transformed after the aggregation.

The HSES dataset also provides information on livestock revenues. Revenues from livestock represent the sales of animals and livestock products. We divide the revenues on the total land cropped to calculate livestock revenues per hectare of land. We also estimate the impact of SDS on vegetation index using normalized difference vegetation index (NDVI). The annual averages of the NDVI data for the study period were obtained from the FAO’s earth data (The data is publicly available at http://www.openforis.org/tools/earth-map.html).

## Results

**Fig 2:**
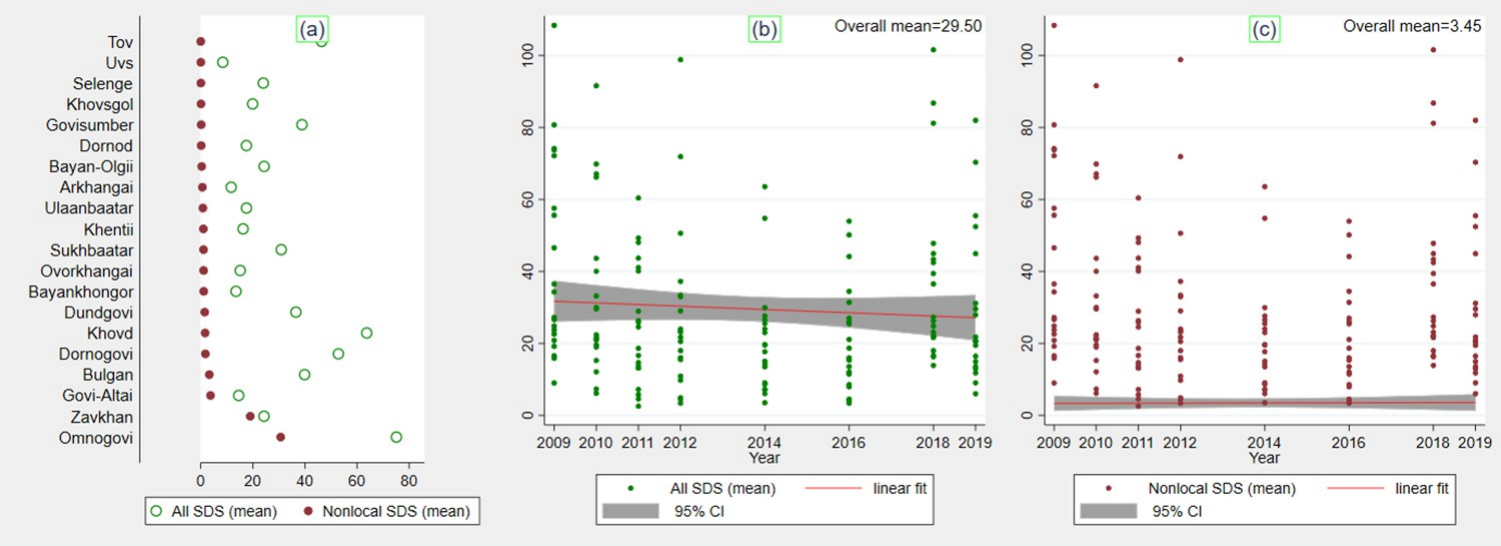
The overall running average of weighted SDS by province and over the years. Source: Authors’ composition based on the MIDAS/CEDA dataset.

We begin by the descriptive statistics of the variables used in the analysis. This is followed by the econometric results from the multivariate regression analysis for each sub-sector. A total of 1,297 dusty days were reported across the country during the period of 8 years (2009–2019). It is worth noting that the HSES data were collected from 2009 to 2019 with gaps; surveys in 2013, 2015, and 2017 were not conducted. Hence the sand and dust storm and climate data for those years were not included in the combined dataset. The summary statistics of all dusty days and non-local dust events over the years and by the province are presented in Table 2 and further illustrated in Fig 2. Omnogovi, Khovd, and Dornogovi were identified as the dustiest provinces, followed by Tov, Bulgan, and Govisumber. The non-local dust emissions show similar distribution, with the exception of Zavkhan, a province that is severely affected by the non-local dust events. The overall trend over time (indicated by the maroon line) in Fig (2B) reveals a slight decrease in all dust events from 2009 to 2019, but the number of non-local SDS appears to have remained more or less the same throughout the eight-year period.

**Table 2:** Descriptive statistics of the variables used in the analysis.

Variable	Obs.	Mean	Std. Dev.	Min	Max
Crop revenue (togrog/ha)	139	8,902,723.5	122,895,91	64,140.18	76,167,424
Potato (kg/ha)	133	12,022.89	14,867.205	400.00	79,439.633
Tomato (kg/ha)	69	871.594	2,178.03	17.963	11,160.715
Onion (kg/ha)	84	563.949	953.35	25.744	5,994.006
Cucumber (kg/ha)	84	473.37	653.05	25.622	3,772.583
Cabbage (kg/ha)	49	4,393.15	6,954.91	616.378	30,601.371
Carrot (kg/ha)	138	1,556.537	2,547.291	1.099	17,246.568
Fruits (kg/ha)	50	1,648.986	3,253.925	24.051	14,000.00
Haylage (kg/ha)	38	4,660.133	4,593.066	159.715	19,607.84
Wheat (kg/ha)	46	6,534.672	13,037.91	116.369	57,817.11
All SDS (days)	160	29.596	22.133	2.553	108.378
Nonlocal SDS (days)	160	3.435	8.126	0.000	43.684
Temperature (C°)	160	1.392	5.024	-24.772	23.673
Precipitation (mm)	160	382.16	258.24	64.22	1769
Agricultural holding (ha)	147	22.651	52.554	0.003	342.99
Agricultural Expenditures (Togrog)	144	4,502,399	6,935,838	89,776.32	39,410,592
Livestock revenue (Togrog)	155	108,074.77	769,010.45	76.534	9,461,035
Animal ownership (all animal)	143	14.117	59.975	.021	510.971
Cattle ownership (cow, sheep, goat)	143	13.33	58.391	.017	497.848
Cow ownership	143	0.729	2.703	0.000	20.398
Animal expenditures(Togrog)	133	16,262.46	21,252.37	0.000	132,517.63
NDVI	160	0.181	0.084	0.064	0.374

The summary statistics for other variables in the analysis, including crop and livestock revenues, crop production, and animal ownership, are also shown in Table 2. Crop revenues are measured in Mongolian Togrog, and quantities of production are measured in kilograms. We divided the total annual production by the total land cropped in that year to estimate crop yields for major crops reported in the survey. Data were carefully inspected to spot potential outliers to ensure the regression estimates are not driven by extreme observations. A box plot of the crop revenues, potato, onion, tomato, and fruits, that showed extreme large values were trimmed at 1% and 5% percentile using *winsor2 –*a package in stata.

The measure of SDS does not account for the duration or the length of the individual events. It only considers the number of events that occurred at a given window of time (e.g., dust days which are then averaged to compute monthly or annual means). The lack of information on the durations of SDS events is a limitation and a challenge in the context of the current study, as this could greatly impact the volumes of airborne particles in the air. Alternative measures from the satellite imagery, such as Aerosol Optical Depth (AOD), do exist, however, AOD is mostly recorded at a specific point in the day, and thus, only provides daily means. Since AOD is not a necessarly continuous measure, it cannot measure the duration of sand and dust events. For instance, AOD observations can only be made during the day, records of nighttime dust storm processes are unavailable [29]. AOD’s are usually measured in a pixel of area (with various resolutions such as 10m, 250m etc.), whereas data on agricultural outcomes are measured at the household, sector, and province level, which presents a similar analytical challenge. Moreover, the AOD measure does not distinguish between external and internal dust events, making it difficult to estimate the endogeneity-net effects of SDS on agricultural outcomes.

Livestock revenues are the sum of gross income that households receive from the sale of animals and animal products. Although the survey reports the ownership of animal by the type of animal, for analytical purpose all animals owned by the household were aggregated; all cattle (cow, sheep, and goat) to see the collective impact of SDS on the ownership of livestock. Multiple regressions analysis were also run for the ownership of cows separately, as the cow ownership is the most important economic activity for the households. The majority of the households in the sample who reported crop production also reported the ownership of livestock.

### Impact on crop production

The baseline fixed-effect regression involving the SDS measure constructed using relevant weather state codes (listed in Table 1) are reported in Table 3. However, caution is advised when interpreting these estimates to avoid misleading conclusions as SDS events are likely endogenous to crop production as discussed in section 2. To account for the potential endogeneity bias in the baseline specification, a variant of the baseline model was run to ensure the estimated results are robust to the potential endogeneity bias. This was accomplished by running the fixed effect regressions of the non-local dust events instead of all events (as indicated by the wheatear state code 06 in Table 1), which are external to local generation of airborne particles. The estimates of this model for most of the crops are largely quantitatively and qualitatively similar (negative) to those of the baseline model, except for the coefficient estimates on SDS for crop revenue, cucumber, and fruit yields that are statically significant in this model but insignificant in the baseline model. This could signify the presence of endogeneity problem in the baseline results masking the true causal impact of SDS. Hence, preference was given to the estimates from the second set of econometric models to control for the endogeneity problem in the estimation by using only non-local dust events. Moreover, standard errors are clustered two-way by province and year to absorb potential correlated shocks across provinces within years. The results from the preferred model are presented in Table 4.

**Table 3:** Impact of SDS on revenues and production based on the baseline model.

	(1)	(2)	(3)	(4)	(5)	(6)	(7)	(8)	(9)	(10)
	Revenue	Potatoes	Haylage	Onion	Cabbage	Cucumber	Wheat	Tomatoes	Fruits	Carrot
All SDS (days)	.008	-.009*	-.035	-.017	0	-.003	-.06***	-.005*	-.022	-.005
	(.008)	(.004)	(.023)	(.01)	(.017)	(.011)	(.013)	(.009)	(.018)	(.009)
Observations	139	133	38	129	49	84	46	108	87	138
R-squared	.644	.628	.498	.578	.328	.459	.688	.437	.435	.614
Time trend	Yes	Yes	Yes	Yes	Yes	Yes	Yes	Yes	Yes	Yes
Year FE	Yes	Yes	Yes	Yes	Yes	Yes	Yes	Yes	Yes	Yes
Aimag FE	Yes	Yes	Yes	Yes	Yes	Yes	Yes	Yes	Yes	Yes

Standard errors are in parentheses

*** p < .01

** p < .05

* p < .1. Standard errors are two-way clustered by the province and year.

**Table 4:** Impact of SDS on revenues and production based on alternative estimation (corrected for endogeneity).

	(1)	(2)	(3)	(4)	(5)	(6)	(7)	(8)	(9)	(10)
	Revenue	Potatoes	Haylage	Onion	Cabbage	Cucumber	Wheat	Tomato	Fruits	Carrot
Non-local SDS (days)	-.027**	-.015***	-.037*	-.034*	-.064*	-.052**	-.244*	-.099**	-.092*	-.030
	(.009)	(.004)	(.017)	(.013)	(.027)	(.019)	(.105)	(.041)	(.058)	(.022)
Observations	139	133	33	129	44	83	39	107	85	138
R-squared	.787	.764	.738	.773	.591	.732	.772	.789	.701	.76
Time trend	Yes	Yes	Yes	Yes	Yes	Yes	Yes	Yes	Yes	Yes
Year FE	Yes	Yes	Yes	Yes	Yes	Yes	Yes	Yes	Yes	Yes
Aimag FE	Yes	Yes	Yes	Yes	Yes	Yes	Yes	Yes	Yes	Yes

Standard errors are in parentheses

*** p < .01

** p < .05

* p < .1. Standard errors are two-way clustered by the province and year.

The results from the preferred estimation approachh revels significantly negative impact of SDS on the value of crop production (gross output) conditional on other covariates (e.g., control variables). There was an estimated 2.7% decline in crop revenue as a result of each additional SDS event. It is worth noting that all the dependent variables throughout this study are log-transformed. According to the World Bank, the total GDP of Mongolia was estimated to be around $14 billion in 2019, to which the agriculture sector contributes about 10% (equivalent to about $1.4 billion). Assuming a steady decline of 2.7% across all agricultural sub-sectors and regions or Aimags, the economic loss from a single dust event could total roughly to $37.8 million, equivalent to about 0.27% of the GDP of Mongolia. However, this estimation would only be accurate and justifiable if an additional dust storm hit every location in Mongolia. Hence, these estimations might not necessarily reflect a realistic extent attributable to a single storm. Furthermore, the regression equation estimates the effect of dust storms as linear in the number of dust storms, hence, extra caution should be used when interpreting these results. This implies that the impact of the subsequent SDS events may not be the same as the first event. This is because first event of dust storm in a season might cause significant damage to crops (i.e., completely root out crops) and therefore the effect of subsequent events would plausibly be less or even zero. Though many assumptions are underlying the analysis, our estimates ofeconomic losses are largely comparable and consistent to the finding of the previous literature assessing the impact of SDS on agriculture. A study that assessed the impact of SDS on the economy of Iran including agriculture sector and estimated 0.04% decline in the GDP of Iran due to a single sand and dust storm event, an amount equivalent to about $149 million [22].

The impact of SDS appear to be negative on crop yields for most crops, with potato, haylage, onion, cabbage, cucumber, wheat, and tomato, experiencing a statistically significant decrease in yields. Although the impact on carrot production is negative, it is statistically insignificant. The impact on wheat is the largest, followed by fruits and vegetables, including tomatoes, cabbage, and cucumber; an additional day of SDS leads to a 24%, 9.2%, and 5–9% reduction in wheat, fruits, and vegetables yields, respectively. The substantially higher losses in wheat are perhaps because dust storms accompanied by strong winds that cause lodging and shattering of wheat crops resulting in higher damages and losses, though this may depend on the growth stage of the crop. Previous literature assessing the impact of dust storms on wheat established similar conclusions. The crop-response model simulations adopted by previous studies suggested that regional haze in China is depressing optimal yields of ≈70% of the crops grown in China, including rice and winter wheat yields by at least 5–30% [30]. The yields of tuber and root crops including potatoes, carrots, and onion are also adversely affected by SDS. The estimated reduction in yields due to an additional event of SDS ranges from 6–8%. Our crop estimations are quite similar to the study looking at the agricultural impacts of SDS on agriculture in Iran that observed a 5–10 percent decline in crop yileds in Iran as a result of single dust storms [22].

Fruits and vegetables (especially vegetables such as cabbage and cucumber which have large leaves) are prone to the accumulation of dust deposition and a subsequent reduction in photosynthesis. Experimental evidence confirms that dust deposition on leaf surfaces induces water stress-like conditions, such as a reduction of stomatal conductance, photosynthesis, transpiration, and increased leaf temperature [31–33]. Another study estimated about 30% reduction in the stomatal conductance and 28% reduction in yield in cotton plants due dust deposition [31]. The impact is most likely even higher on leafy vegetables.

SDS were also found to have a statistically significant affect on haylage production including different forages; with an additional day of SDS reducing the haylage yields by about 4%. Potato production, on the other hand, is not as severely affected, with an additional day of sand and dust storm resulting in about 1% reduction in the potato yields. Although the impact on carrot production is negative, it is not statistically significant. These findings are plausible, as tuber and root crops are not likely to be as susceptible to dusts and wind speed.

**Fig 3:**
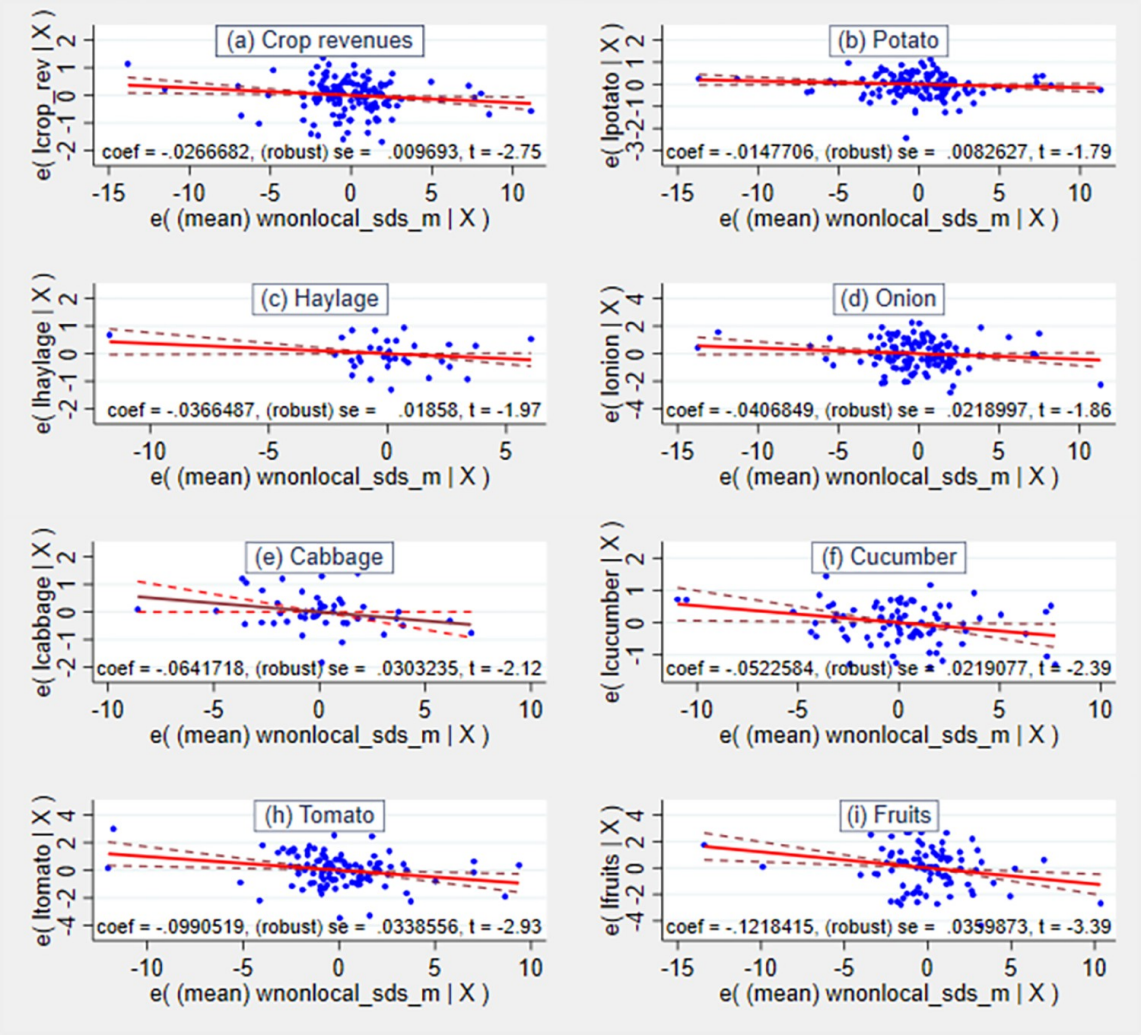
Added-variable plots of the correlation between an SDS conditional on other covariates. Source: Authors’ composition based on the HSES and MIDAS/CEDA datasets.

Fig 3 illustrates the estimated negative effects of SDS on the value of crop production and physical yields conditional on other control variables. The fitted regression line showing the relationship between these transformed variables has the same slope as the coefficient on sand and dust storm variable in the full regression model which includes all the control variables. In other words, the fitted line shows a partial correlation between the independent and dependent variable. Hence, the impact is represented by the estimated coefficient which corresponds to the slope of the fitted line (indicated by red color). The conditional mean of revenue and yields are strictly decreasing with a raise in the number of dust events conditional on the control variables including climate (precipitation and temperature) and household socioeconomic characteristics, such as the agricultural holding and expenditures on agricultural inputs.

### Impact on livestock

Dust storms affect the livestock sector in several ways. The impacts include direct physical harm from stress to their physical environment and health, which thereby leads a reduction in animal products such as dairy and meat as well as higher animal mortality rates. Moreover, lost, destroyed, or damaged pasture or forage crops increase the financial burden of animal husbandry, as households must purchase additional animal feed that otherwise would not been required [11]. Econometric results from this analysis confirm the adverse effect of SDS on the livestock sector. The results in Table 5 reveal a significantly negative impact of SDS on the value of livestock production (gross income); each additional SDS event decreases livestock revenues by 3.2%. Livestock revenues comprise of revenues associated with the sales of animals and animal products including dairy, wool, skin etc., which implies a direct loss of the animals due to mortality and a decline in the animal production.

**Table 5:** Impact of sand and dust storms on livestock revenues and ownership.

	(1)	(2)	(3)	(4)
.	Revenue	All animal	Cattle	Cow
Nonlocal SDS (days)	-.032*	-.048**	-.05**	-.059***
	(.01)	(.016)	(.015)	(.021)
Observations	109	130	130	124
R-squared	.69	.803	.793	.809
Time trend	Yes	Yes	Yes	Yes
Year FE	Yes	Yes	Yes	Yes
Aimag FE	Yes	Yes	Yes	Yes

Standard errors are in parentheses

*** p < .01

** p < .05

* p < .1. Standard errors are two-way

clustered by the province and year.

Besides the negative impacts on earning potentials, livestock ownership decreases with increasing SDS. The reduced animal ownership could be attributed to the direct loss of animals (high mortality), thus disincentivizing household ownership of the animals, especially in areas where SDS occur more frequently. It can also be explained by the physical damages to the animal structures and decline in forage production, as was hypothesized in the literature [11]. A study using a cross-sectional survey from livestock owners to have assessed the long-term impact of SDS on livestock quality of life and mortality, concluding that SDS cause significant animal losses and long-term negative impacts on their quality of life [34]. It is also worth noting that livestock ownership may be declining because of other reasons, such as households butchering animals for sale and consumption, or selling them to herders in neighboring Aimags who might be less affected by dust events, hence caution should be taken in interpreting these results.

### Impact on land use and vegetation coverage

In addition to the impacts of SDS on crop and livestock sectors, causal links between sand and dust storm and land use changes and vegetation cover (ground biomass) were also investigated. Land under cultivation (e.g. area cropped) decreases with the increase in the number of SDS events; that is, households cultivate less land to adjust for the negative consequences of SDS. Holding all other variables constant, a 6% decrease in land cultivation can be attributed to each SDS event (Table 6), a relatively large impact for land owners in Mongolia, where land holding sizes are considerably small. The impacts of SDS on the households’ land use and planning decisions could be attributed to the reduction in crop yields and productivity (as observed in the previous sections) as well as the erosion of the soil, which could force farm households to abandon previously productive farmlands. The productivity effects may render crop production to be less remunerating which would ultimately effect households’ decisions related to land allocation. It’s also worth noting that some of the cropping decisions for the current year may have been made in the previous year, or that dust storms early in the year may have influenced cropping selections for later in the year. The data does not allow to distinguish interannual variations in land allocation decisions, however the time fixed effects in the model capture such variations to control for the average differences across time.

**Table 6:** Impact of sand and dust storms on land use and vegetation cover (ground biomass).

	(1)	(2)
	Land use	NDVI
Nonlocal SDS (days)	-.064**	-.001**
	(.026)	(.000)
Observations	147	147
R-squared	.481	.98
Time trend	Yes	Yes
Year FE	Yes	Yes
Aimag FE	Yes	Yes

Standard errors are in parentheses

*** p < .01

** p < .05

* p < .1.

Standard errors are two-way clustered by the province and year.

The SDS appears to have a negative but very small impact (0.1%) on the normalized difference vegetation index (NDVI). SDS leads to significant desertification in agricultural and pastoral lands. Previous literature on Mongolia confirms the adverse effects of SDS on desertification and ground biomass, especially in the ecologically fragile regions. Using a long term observational data to assess variation trends of dust storms in relation to meteorological conditions, a recent study found significantly negative correlation between sand and Dust storm and the NDVI index in Mongolia [35]. Similarly, a cross-country analysis in Central Asia, North China, and Mongolia assessing the statistical relationship between environmental factors, vegetation, sand and dust storm frequency and concluded that vegetation condition was negatively correlated with the SDS frequency, while dryness and the SDS frequency were positively correlated [36]. Broader literature also link the adverse impacts of SDS and vegetation cover in Iran, Iraq, and Kuwait [37–39].

## Discussion and concluding remarks

Despite the growing concerns about the potential effects of SDS on socioeconomic factors, including health, transportation, and agriculture, little empirical evidence exists to quantify their economic losses and inform policy. This paper presents empirical evidence analyzing the causal links between dust exposure and agricultural productivity in Mongolia. Our analysis spans historical household-level socioeconomic and climate data allowing for exploration of the effects of SDS on crop productivity, physical yields of various crops, land use, and vegetation cover in Mongolia. The analyses reveal compelling evidence SDS has significant bearings on crop and livestock productivity, land use planning, and vegetation coverage.

We find about 3% and 3.2% decline in the value of crop and livestock production (gross income) as a result of an additional event of SDS. Assuming a constant decline of 2.7% across all agricultural sub-sectors, the loss of a single dust event could equate to about $0.37.8 million, equivalent to about 0.27% of the GDP of Mongolia. Previous research establishes similar findings in Iran and West Africa. We extend our analysis to assess the impact of SDS on the physical production of several crops. The impact of SDS appear to be negative on crop yields for most crops, with potato, haylage, onion, cabbage, cucumber, wheat, and tomato, experiencing a statistically significant decrease in yields. The impact on wheat is the largest, followed by fruits and vegetables including tomatoes, cabbage, and cucumber; an additional day of SDS leads to a 24% 12%, 5–9% reduction in wheat, fruits, and vegetables yields, respectively. The significantly higher losses in wheat are perhaps attributable to SDS and its accompanied strong winds, which cause lodging and shattering of wheat crops and thus higher damages and losses, although this may depend on the growth stage of the crop. The leaves of fruits and vegetables (especially vegetables such as cabbage and cucumber which have large leaves) are prone to the accumulation of dust deposition which reduces photosynthesis and stomatal conductance, thereby hindering biomass production.

A variant of baseline specification using an alternate measure of sand dust storms involving dust events from non-local sources alone was estimated to test and ensure that the estimated impacts are robust, especially given the potential estimation bias due to endogeneity in the SDS measure. Storms origionating from from external soures are likely to be external to local production. To effectively control for endogeneity bias and obtained unbiased results, ideally, instrumental variables are needed to break the unexplained relationship between SDS and the error term, a relationship that may arise due to reverse causality or omitted variable bias. However, the dataset used in the analysis lacks the variables that could have potentially serve as instrumental variables in this analysis. It is recognized as a limitation and a proposed a workaround is the construction of an alternative measure of the SDS involving events that are originated from external sources (instead of the measure involving dust from all sources used in the baseline specification).

These findings reveal that SDS pose a critical threat to agriculture, food security, environment, and sustainable development. The analyses of SDS suggest that these events have critical implications for policy in the affected countries and regions. To achieve the dual objective of maintaining crop productivity and restoring degraded land and environmental resources, national and regional agricultural policies must recognize the increasingly damaging effects of SDS to crops, livestock, and the environment.

Experts advocate that future policies will need to evolve around a two-fold framework to mitigate the hazards associated with SDS [2, 5], with “impact mitigation” and “source mitigation” as the key elements of the proposed approach. “Impact mitigation” through integrated early warning and monitoring, risk/impact assessment, and vulnerability mapping of at-risk populations and infrastructure can help increase awareness in their communities to better prepare and adjust for the potential impacts of SDS. “Source mitigation” involving sustainable land and landscape and integrated water management can help reduce the frequency and occurrence of SDS. While mitigation tools could pose higher economic costs, adaptation strategies could be warranted in the context of agriculture on the grounds of their cost-effectiveness and practicality. Resilience building through adaptation strategies could help effected farm household better cope with the negative consequences of natural hazards.

Future strategies for mitigating the adverse effects of SDS on agriculture need to be participatory, tailored to the context, and centered on community-based approaches that integrate effective crop and land management practices, while promoting soil conservation measures at the national level. Prior literature indicated that past efforts to combat SDS have met with limited success because of lack of involvement of the affected communities in planning and implementing the strategies [40]. With that in mind, future strategies will therefore need to be different in target and scope to effectively support effected agricultural communities.

Given the complex nature of SDS and their transboundary effects, there is increasing interest in regional-scale policies to combat SDS. These transboundary polices can be implemented by individual countries through national action plans. Indeed, countries could join forces to combat SDS in collaboration to achieve higher impacts at the regional level. Recently the United Nations General Assembly (UNGA) adopted resolutions entitled “combatting sand and dust storms” in 2015 (A/RES/70/195) and 2016 (A/RES/71/219). Following these resolutions, the United Nations Convention to Combat Desertification (UNCCD) has been working to deploy regional-scale policies to assist member countries in developing more proactive policies with better predictive mechanisms to combat SDS and mitigate their effects on human life and economic activities.

## Supporting information

S1 File Data and stata do files. (ZIP)
